# A biophysical model of endocannabinoid-mediated short term depression of excitation in hippocampus

**DOI:** 10.1186/1471-2202-14-S1-P66

**Published:** 2013-07-08

**Authors:** Margarita Zachariou, Chris Christodoulou

**Affiliations:** 1 Department of Computer Science, University of Cyprus, Nicosia, 1678, Cyprus

## 

Cannabinoids are a group of chemical substances that activate cannabinoid receptors. The general types of cannabinoids are (1) phyto-cannabinoids, naturally occurring in the cannabis plant, (2) synthetic cannabinoids, produced in the laboratory, and (3) endocannabinoids which naturally form endogenously in the body. The endocannabinoid signalling system is known to have a neuromodulatory role in many physiological processes including neurotransmission, synaptic plasticity, learning and memory [1]. Endocannabinoids are synthesised in response to neuronal activity in a calcium-dependent manner and/or due to G protein-coupled receptor activation. Once released into the synaptic cleft, they act in a retrograde fashion on pre-synaptic cannabinoid receptors resulting to inhibition of neurotransmitter release. Endocannabinoids are known to underlie certain forms of short- and long-term forms of synaptic plasticity at both excitatory and inhibitory synapses in various brain areas [1]. In hippocampus, a form of endocannabinoid-mediated short-term depression (eCB-STD), which is calcium-dependent, is known as depolarization-induced suppression of inhibition (DSI) or excitation (DSE) [3]. Both hippocampal DSI and DSE can be induced by application of cannabinoid receptor agonists and are blocked by cannabinoid receptor antagonists [2,4]. Although DSI and DSE in hippocampus share many common biochemical signalling pathways, DSE is less prominent than DSI and requires longer depolarisations for induction supposedly due to the lower expression and sensitivity of cannabinoid receptors on excitatory cells [3]. However, the expression of cannabinoid receptors on excitatory terminals is more homogeneous than in inhibitory terminals (only about 50% of inhibitory terminals are sensitive to eCB-STD) [3].

Here, we present the development of a mathematical model of the endocannabinoid signalling underlying DSE at hippocampal excitatory synapses on excitatory cells which is (a) biophysically plausible, describing the most essential pathways underlying this phenomenon (b) detailed enough to describe the key DSE experimental characteristics, and, (c) is simple enough to use in network studies. The main components of the DSE model are (i) a post-synaptic single compartment model for the excitatory cell with endocannabinoid synthesis mediated by intracellular calcium dynamics, and, (ii) a pre-synaptic single compartment model for the excitatory cell with cannabinoid receptor dynamics modulating excitatory synaptic transmission. As a modelling framework for the cannabinoid signalling we consider a recently developed biophysical model for endocannabinoid-mediated short-term depression of inhibition in hippocampus [5]. We extend and adapt this model in order to account for the reduced magnitude and higher induction threshold for DSE versus DSI. The model successfully reproduces the mediation of DSE due to endocannabinoid-mobilising stimulation (depolarising voltage pulses), as observed experimentally [3]. The model also successfully captures the mediation of endocannabinoid-mediated short-term depression of excitation due to cannabinoid agonist administration (WIN55,212) [3]. The development of the DSE model opens the way for comprehensive networks studies including both DSI and DSE, in order to decipher the role of cannabinoids in synaptic transmission in network emergent behaviour related to memory and learning.
